# Cerebral Venous Sinus Thrombosis Complicating Herpes Zoster Ophthalmicus Ophthalmoplegia

**DOI:** 10.7759/cureus.56520

**Published:** 2024-03-20

**Authors:** Meithem Ali, Nonyelum Obiechina, Kay Teck Ling, Angela Nandi, Bhaskar Mukherjee

**Affiliations:** 1 Acute Care Common Stem, NHS England Kent, Surrey and Sussex, Kent, GBR; 2 Geriatric Medicine, Queen's Hospital Burton, Burton-on-Trent, GBR; 3 Geriatric Medicine, Sandwell and West Birmingham NHS Trust, Birmingham, GBR

**Keywords:** herpes-zoster virus, herpes-zoster ophthalmicus, cerebral venous sinus thrombosis (cvst), ophthalmoplegia, headache

## Abstract

Cerebral venous sinus thrombosis (CVST) is a rare cause of strokes and is most common in younger patients particularly those less than 50 years of age. It is more common in females than in males and is known to be associated with pregnancy, puerperium, oral contraception, congenital and acquired thrombophilia, and malignancy. Less commonly, it has been shown to be associated with infections and more recently has been found to be associated with COVID-19 infection with thrombocytopenia and the COVID-19 vaccine AstraZeneca. Rare cases have been reported in association with varicella zoster virus (VZV) infection (chickenpox) and its reactivated version of herpes zoster virus (HZV) infection (shingles). We report the case of a 68-year-old lady with herpes zoster ophthalmicus ophthalmoplegia who developed cerebral venous thrombosis (CVT).

## Introduction

Cerebral venous sinus thrombosis (CVST) is a rare condition in which a thrombus forms in the dural venous sinuses in the brain [1]. It frequently occurs in prothrombotic conditions with hypercoagulability and damage to the sinuses and veins, leading to impaired venous drainage, raised intracranial pressure, focal neurological symptoms, and seizures [1]. Although the increased hypercoagulability of pregnancy and puerperium is its most common known association, oral contraceptive pill (OCP) and hormone replacement therapy (HRT), trauma, cancer, and infections may also play a role in its genesis [1]. Rarer associations have been described between CVST and varicella zoster virus (VZV) infection or chickenpox and herpes zoster virus (HZV) infection or shingles.

Proposed hypotheses for this association include increased inflammatory response to the infection with subsequent vasculitis [2]; others suggest increased hypercoagulability due to immunological mimicry of protein C and protein S by blocking antibodies following infection with VZV or HZV [2].

Herpes zoster ophthalmicus (HZO) occurs when there is HZV involvement of the first branch (ophthalmic branch or V1) of the trigeminal nerve or fifth cranial nerve (V1) [3]. It tends to give a vesicular rash over the forehead on the same side. It may also cause keratitis and uveitis if the nasociliary branch of the ophthalmic nerve has a rash of the nose and tip of the nose on the same side (Hutching's sign) [3]. If HZV infection is not treated early in these patients, there is an increased risk of visual impairment and blindness.

Ophthalmoplegia may also occur due to the contiguous involvement of surrounding tissues by inflammation, leading to myositis and perivasculitis of the ocular muscles, frequently the third, fourth, and/or sixth cranial nerves either in isolation or in combination [3]. Here, we present a case of HZO ophthalmoplegia due to third cranial (oculomotor) nerve palsy and CVST in a 68-year-old lady.

## Case presentation

A 68-year-old Caucasian female initially presented to our district general hospital emergency department in March 2021 with a sudden onset of sharp, stabbing left-sided temporal headaches that had developed a week previously. It was associated with left periorbital pain and pain in her left earlobe. The headaches were constant and not associated with any aggravating or relieving features.

She attended the emergency department because she noticed a red rash on her left forehead the day before. She did not have any blurred vision or any other visual symptoms at that point. She had a past medical history of ocular hypertension in both eyes and asthma but no other history of note. She was not on any regular medications. She had a family history of glaucoma but did not have any other significant family or social history. She was found to have a red, vesiculopapular rash over her left forehead but no focal neurological features or papilledema. Her general and systemic examinations were all normal. A diagnosis of HZO was made, and she was treated with oral acyclovir 500 mg five times daily and reviewed by the Eye Department who prescribed topical acyclovir and chloramphenicol eye drops for her. She was discharged with a follow-up appointment with the Eye Department.

Four days later, she developed double vision and some drooping of her left eyelid. She was seen by her general practitioner who referred her to the emergency eye clinic. She was found to have a left third cranial nerve palsy with complete left ptosis and pupillary sparing. She had ophthalmoplegia with impaired adduction, elevation, and depression of the left eye. Downward and inward movement and abduction of the left eye were preserved. There was no evidence of keratitis or uveitis, and Hutching's sign was negative. There was no evidence of papilloedema. The ophthalmologist made a diagnosis of HZO ophthalmicus with third cranial nerve palsy and referred her urgently to the district general hospital emergency department. 

On clinical examination, she was apyrexial with a normal pulse rate, which was regular, and a normal blood pressure. Her chest, cardiovascular, and abdominal examinations were unremarkable. She had a vesicular rash over the left forehead with evidence of a left third cranial nerve palsy with complete ptosis and partial ophthalmoplegia. She did not have any other focal neurological signs. The rest of her clinical examination was unremarkable. Preliminary investigations revealed that her full blood count, urea, electrolytes and creatinine, erythrocyte sedimentation rate (ESR), and c-reactive protein (CRP) were normal as were her chest X-ray and computerized tomography (CT) scan of her head. A diagnosis of HZO with oculomotor palsy (HZO ophthalmoplegia) was made.

She was initially treated with intravenous acyclovir 10 mg/kg and given analgesics. Unfortunately, her headaches persisted, and she had a vasculitis screen and a screen to exclude conditions that cause immunosuppression, such as human immunodeficiency virus (HIV). They all came back negative. It was felt that she did not have giant cell arteritis (GCA) as her ESR and CRP were normal.

**Figure 1:**
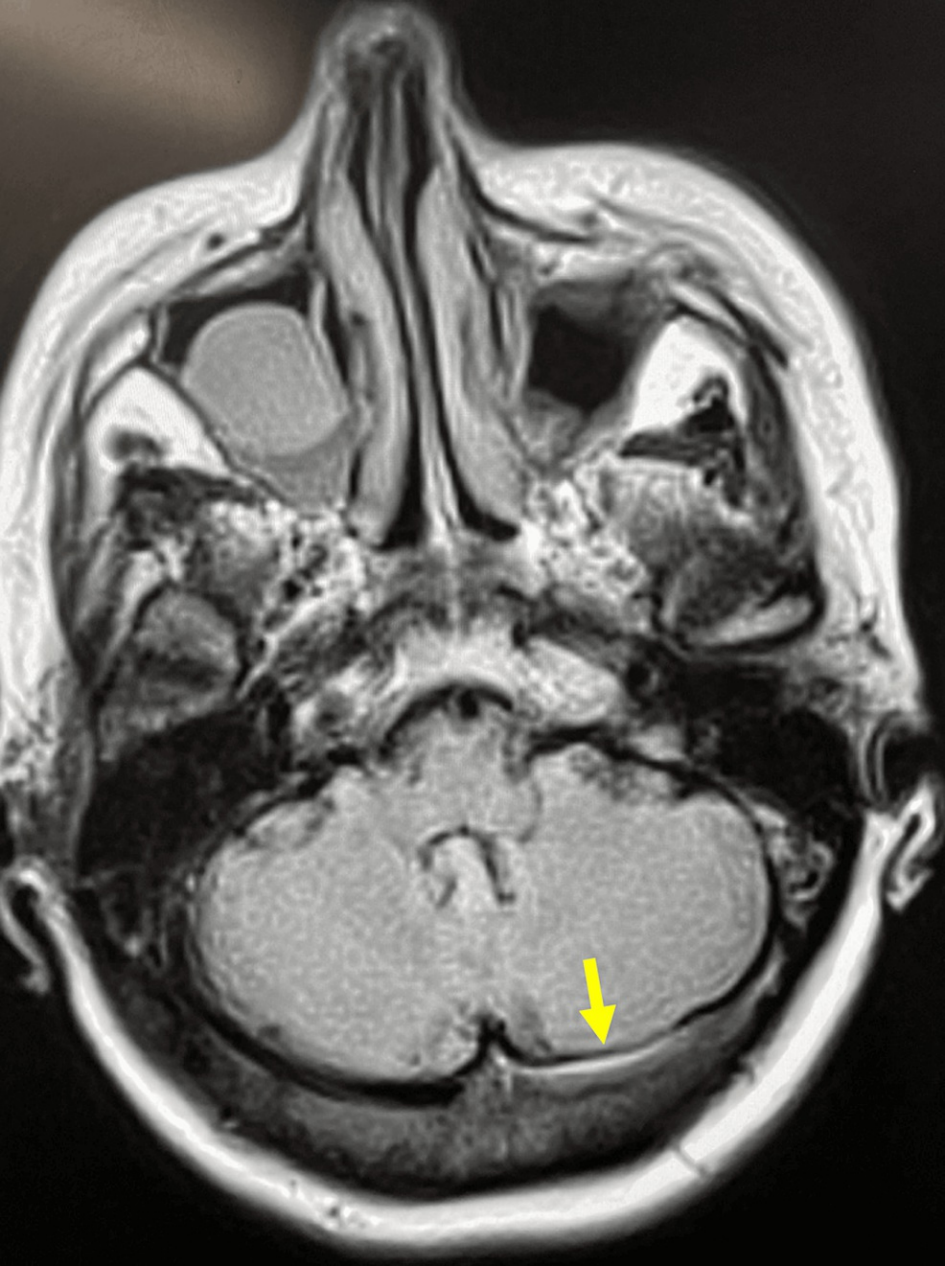
MR imaging with features indicating CVST. MR: magnetic resonance, CVST: cerebral venous sinus thrombosis

**Figure 2:**
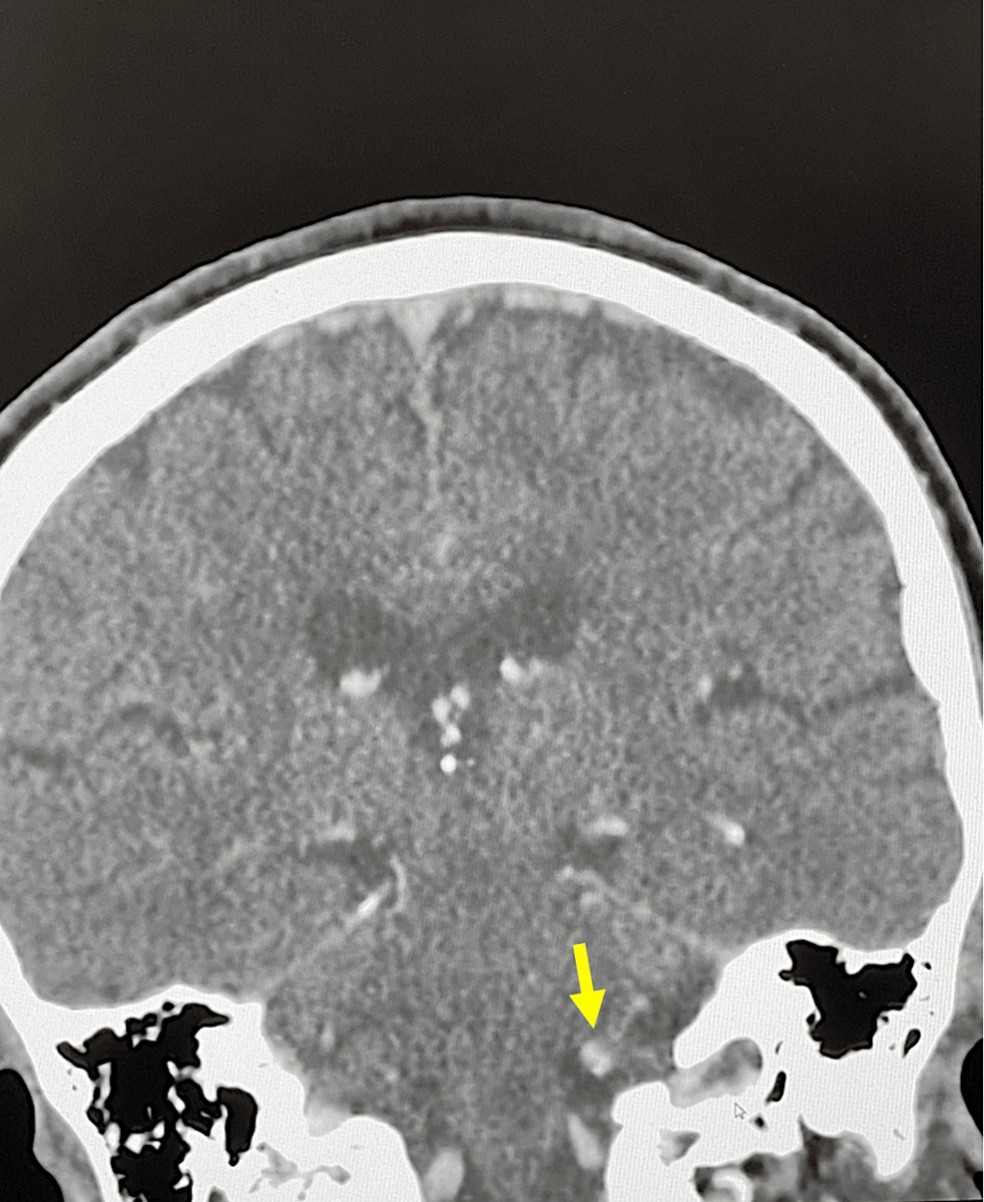
CT venogram demonstrating CVST with involvement of the left sigmoid sinus and left internal jugular vein. CT: computed tomography, CVST: cerebral venous sinus thrombosis

She did not have a temporal artery biopsy as these can also show skip lesions. The ophthalmologist felt that she did not have giant cell arteritis. She had a magnetic resonance imaging of the arteries (MRA) to see if she had an aneurysm, such as a posterior communicating artery aneurysm. It did not show an aneurysm but rather showed features suggestive of CVST (see Figure 1). This was further confirmed on a CT venography (CTV), which showed a non-occlusive thrombus in the left transverse and sigmoid sinus extending into the left internal jugular bulb (see Figure 2). As mentioned above, her full blood count was normal and did not show any evidence of thrombocytopenia. She had tested negative for COVID-19 on polymerase chain reaction (PCR). Although she had received the COVID-19 vaccine AstraZeneca several weeks before, her platelet count was normal with no evidence of thrombocytopenia. The hematology team also felt that this was unlikely to be due to her COVID-19 vaccine. A diagnosis of CVST probably related to her HZO was made. Following a discussion with the neurology team, she was commenced on low-molecular-weight heparin (LMWH) enoxaparin 1.5 mg/kg and warfarin. Unfortunately, she had already been commenced on anticoagulants before her thrombophilia screen was done. She was seen in the hematology clinic after discharge and had her anti-thrombin III done, which was negative. Her antiphospholipid antibody also came back negative. Because she remained on warfarin her protein C and protein S levels could not be done.

Progress and outcome

**Figure 3:**
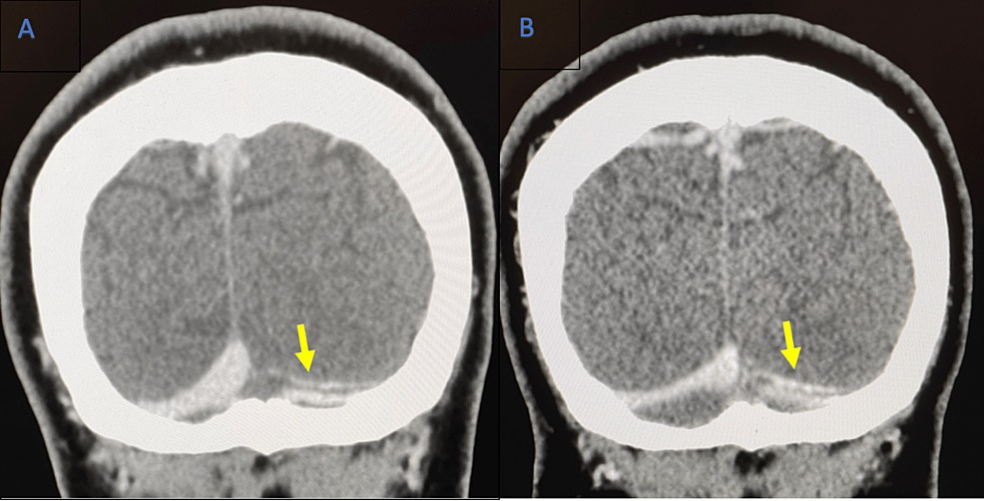
CT venogram demonstrating CVST in the left transverse sinus A: CT venogram at diagnosis, B: CT venogram six months after therapy.

Her headaches improved as did her ptosis, but this made the diplopia more evident. She was followed up several months later by the ophthalmology team and her ophthalmoplegia and diplopia had largely resolved. She was treated with warfarin at a target international normalized ratio (INR) of 2 to 3. After six months of treatment, she had a repeat CTV (see Figure 3B), which showed no significant change from her previous CTV (see Figure 3A). Because of the persistence of thrombi in the venous sinuses, a further follow-up in the hematology clinic has been scheduled to discuss further options. In the meantime, she continues on warfarin.

## Discussion

CVST is a rare type of cerebrovascular disease affecting the superficial and deep cerebral veins and cerebral dural venous sinuses [1]. It accounts for about 1 to 2 percent of all strokes and tends to occur in the younger age group than arterial strokes [1]. It was first described by Ribes, a French physician, in 1825 in a 45-year-old patient who had presented with delirium, headache, and seizures [4]. An autopsy performed after his death showed superior sagittal sinus and lateral sinus thromboses [4]. Since then, a better understanding of the aetiological, epidemiological, and pathophysiological elements of CVST has led to effective treatment options [5]. CVST is commonly associated with pregnancy, puerperium, inherited and acquired thrombophilia, use of contraceptive pills, and hormone replacement therapy (HRT) [6]. In rarer cases, it may be associated with viral and /or bacterial infections and malignancies, such as myeloproliferative disorders [7]. More recently, it has been found to be associated with COVID-19 infection with attendant thrombocytopenia [8]. Some concerns have also been raised about cases of CVST with thrombocytopenia following COVID-19 vaccination with AstraZeneca [8]. Although not widely known, CVST associated with VZV and HZV has been reported in several case reports [9-14].

VZV is a member of the alpha herpes species of viruses [15]. It frequently causes an itchy vesicular rash in children called chicken pox, and most children would have developed immunity to VZV by the time they are 12 years old [15]. The infection can occur in adults and, more rarely, in older patients for the first time, and the presentation is a lot more severe [16]. Its manifestations in these late presentations may include encephalitis, aseptic meningitis, Guillaine-Barre syndrome, and pneumonitis [16]. However, a majority of people have developed IgG antibodies to chickenpox by the time they are 65 years of age [15,16]. Following a primary infection, the VZV migrates by retrograde conduction up the axons of dermatomes or cranial nerves to the posterior nerve root ganglia, autonomic ganglia, and cranial nerve ganglia [17]. It downregulates its DNA, reduces the activity of the major histocompatibility complex (MHC), and goes into a dormant state [17]. During periods of immunosuppression, the virus is able to over-ride the body’s immune system, migrate down the axon, and cause vesicular eruptions in the area innervated by the nerve [17]. When this process, called latent reactivation, occurs, the virus is referred to as HZV, and the lesions are called shingles. It can result in overwhelming, generalized systemic infections in immunosuppressed patients, but it more commonly causes a unilateral dermatomal vesicular lesion in the area sub-served by the relevant nerve [17]. In some cases, it causes neuritis and neuropathic pain or post-herpetic neuralgia [17,18].

When it affects the cranial nerves, it can result in nerve palsies [17,18]. In the case of this patient, she developed an HZV infection involving the first branch (ophthalmic nerve) of the trigeminal nerve (fifth cranial nerve) or V1. The infection, also known as HZO, accounts for about 20 percent of all patients with herpes zoster infection or shingles [17,18]. HZO frequently causes vesicular lesions on the forehead in the ophthalmic nerve branch territory [17,18]. When its nasociliary branch is affected, it gives rise to vesicular lesions at the tip of the nose (Hutchin’s sign) [18,19]. In these instances, it is more likely to result in serious eye infections, such as pseudokeratitis, uveitis, and in some cases retinal detachment [19]. Other cranial nerves, such as the third (oculomotor), fourth (trochlear), and sixth (abducens) cranial nerves, may become involved as a part of neuritis, which can complicate the HZO although this is relatively rare [19]. The oculomotor nerve is the most commonly affected cranial nerve, as occurred in this patient, and can result in pupillary dilatation, complete ptosis, and ophthalmoplegia [19]. In very rare instances, all the aforementioned cranial nerves are affected at the same time as a part of a condition called the orbital apex syndrome (OAS) [19]. OAS is a rare condition characterized by the involvement of the second, third, fourth, and sixth cranial nerves in addition to the ophthalmic branch of the fifth cranial nerve (V1). Its aetiological factors include trauma, inflammation, infections, and malignancies [19]. It is very rarely due to HZO, and in these instances, immunological reaction to the virus has been implicated as one of the mechanisms by which this may occur [19].

VZV and HZV have been linked to vasculopathy and arterial strokes and, less often, venous strokes, such as CVST and cerebral venous thrombosis (CVT) [14,20]. The mechanisms behind this association are not well understood [18]. However, acquired protein S deficiency due to the VZV inducing autoantibodies to protein S has been proposed as a possible explanation [20]. Damage to the vascular endothelium with stasis of blood is another hypothesis [20].

Patients with CVST may either not be diagnosed or diagnosed late due to the non-specific features of its presentation [21]. A high degree of suspicion is therefore necessary to prompt the relevant radiological investigations to confirm it. Clinical features of CVST include headaches, which may occur in up to 80 % of cases; it may be unilateral occurring on the affected side or it may be bilateral [1,21,22]. Depending on which venous sinus is involved, features such as hemiparesis, monoparesis, or seizures may also occur [21,22]. In extreme cases, coma and death may occur [21,22]. Death is usually caused by raised intracranial pressure with transtentorial herniation. Diagnosis is frequently made on CTV or MR venogram (MRV) [1,22]. Looking for underlying causes, such as screening, for underlying malignancy and thrombophilia is an important part of investigating these patients [1,22]. Unfortunately, our patient was quickly commenced on anticoagulants before blood had been taken for a thrombophilia screen. 

Treatment in the acute stages is usually with LMWH, followed by warfarin, which can be continued for three to six months [1,22]. The duration may be longer in unprovoked CVST and recurrent CVST [1,22]. Direct oral anticoagulants (DOACs) are still being evaluated at present in CVST and so are not currently licensed for use in this context [1,22]. Bleeding is a potential side effect of anticoagulation but has to be balanced against the risk of clot propagation, worsening of the CVST, and recurrence of the CVST [1,22]. Where anticoagulation is contraindicated or patients’ symptoms are not improving, alternatives such as endovascular thrombolysis or thrombectomy in specialist centers may need to be considered [1,22]. It is also important to identify and treat the underlying cause and manage complications such as seizures and raised intracranial pressure with anti-epileptics and intravenous mannitol, respectively [1,22]. In the case of our patient, she had already received acyclovir for her HSV infection and had been reviewed by the ophthalmology team for her third cranial nerve palsy with ophthalmoplegia. In extreme cases with large hemorrhagic infarction due to CVST, decompressive craniotomy may be used as the last resort [1,22].

The prognosis of CVST is generally favorable when it is identified early and treated [1,22]. Late diagnoses may lead to increased mortality and residual neurological deficits not dissimilar to those of arterial strokes [1,22]. This underscores the need for a high index of suspicion given the frequently nonspecific initial presentation of this condition, as in our patient.

## Conclusions

CVST is a rare but potentially treatable type of stroke that tends to occur mainly in younger females. Its presentation can be non-specific, thereby delaying diagnosis, and this can lead to death or severe, permanent, neurological damage. Although it is relatively rare in VZV and HZV infections, CVST can occur and should be sought in the presence of unexplained headaches or other neurological findings in a patient with HZO.
